# Cranial Sutures Alter Computational Models of Transcranial Electrical Stimulation

**DOI:** 10.1097/YCT.0000000000001154

**Published:** 2025-05-02

**Authors:** Alistair Carroll, Caroline Rae, Donel Martin, Socrates Dokos, Colleen Loo

**Affiliations:** * Discipline of Psychiatry, University of New South Wales, Sydney Australia; † University of New South Wales, Sydney, Australia; ‡ Discipline of Psychiatry, University of New South Wales, Sydney, Australia; § Graduate School of Biomedical Engineering, University of New South Wales, Sydney, Australia

**Keywords:** cranial sutures, transcranial electrical stimulation, computational modeling, ROAST

## Abstract

**Background:**

ROAST (Realistic vOlumetric-Approach-based Simulator for Transcranial electric stimulation) has been increasingly utilized to inform studies of transcranial electrical stimulation (tES). The precision of ROAST is dependent on anatomical accuracy.

**Objectives:**

The aims of the study were to compare using only T1 magnetic resonance images in ROAST (T1 model), with a combination of T1 and T2 magnetic resonance images (T1 and T2 model) and to model the electrical fields generated by tES with commonly used ECT montages: bitemporal, bifrontal, and right unilateral and varying the skull conductivities (based on the electrode position) and including cranial sutures.

**Methods:**

The “T1 model” was selected for computational modeling. The skull conductivity was varied from the “default setting in ROAST” (0.01S/m) to that of temporal bone (0.0038S/m) and frontal bone (0.0126S/m). “Disc” electrodes (5 cm in diameter) were applied with 1 mA of current. “Pad” electrodes, 0.1 mm high and 40 mm wide, were positioned over the squamous suture, coronal suture and sagittal suture and the skull conductivity changed to approximate suture conductivity (0.32S/m).

**Results:**

The “T1 model” differed from the “T1 and T2 model,” which resulted in variable electric fields reaching individual tissue layers. Changing skull conductivity and simulating the cranial sutures resulted in changes to the electric current reaching the cortical and subcortical structures with the later having a greater impact.

**Conclusions:**

This study demonstrates the importance of anatomically accurate head models in ROAST computational modeling of tES. Varying the skull conductivity based on studies in vivo and including cranial sutures is imperative for more realistic predictions of electric field and reproducibility.

Transcranial electrical stimulation (tES) includes electroconvulsive therapy (ECT) and transcranial direct current stimulation (tDCS). ECT involves the administration of pulsed electrical current to induce generalized seizures, whereas tDCS induces a constant, low intensity, unidirectional current. Both are used clinically to treat major depressive disorder and schizophrenia.^1–3^

Over the last decade, there has been an evolution of computational models of transcranial current stimulation.^4^ Early modeling required manual segmentation of the various head tissues from magnetic resonance images (MRIs) and conversion into a finite element mesh then simulation of the electrical current from manually positioned electrodes.^5^ Open-source pipelines have now been developed that automate this process and have reduced the time to generate a computational model. This has increased the opportunity for studies to use them; a recent review documented 94 clinical tES studies that have leveraged one of these pipelines, “ROAST,” to make over 1800 head models for more than 30 different clinical indications.^6^ It was not possible to perform tES modeling studies with rigor and reproducibility before pipelines such as ROAST (Realistic vOlumetric-Approach-based Simulator for Transcranial electric stimulation) were created. Theoretically, automating the process reduces human subjectivity from manual segmentation and improves reproducibility.

A limitation of the emergence of multiple different pipelines is that the segmentation procedure differs, which leads to variable results. ROAST uses a volumetric approach by using a segmentation algorithm that works on voxel image data from MRI images and can create models in less than 30 minutes. ROAST represents the skull as one layer and portrays skull details very accurately and small external features such as cranial sutures can be visualized, however, cannot be added to the automated pipeline with a different conductivity to the skull. In contrast, another popular pipeline, SimNIBS, uses surface mesh segmentation of the MRIs images and can segment the skull into 3 layers and allows the user to add masks to represent pathology or anatomical features such as the sutures.^4^ A study demonstrated that the different methods used by ROAST and SimNIBS resulted in a divergence of up to 47% (over 0.1 V/m) in the calculated electric fields in brain and CSF from tDCS simulation using the F3-F4 montage.^7^

Improving the anatomical accuracy of head models would theoretically result in more realistic and accurate results. A study that took 1.4-cm bone plugs from 48 different skull flaps from patients undergoing surgery for epilepsy demonstrated that skull conductance increases proportionally to the % of spongiform bone and skull thickness.^8^ The frontal bone is thicker than the temporal bone and has a larger proportion of spongiform bone, which results in a higher conductivity of 0.0126 S/m compared to 0.0038 S/m for the temporal bone. These differ from the default skull conductivity used in the ROAST modeling of 0.01S/m. A meta-analysis including 20 studies and 121 subjects measured the weighted mean skull conductivity as 0.016 S/m with standard deviation of 0.019 S/m and concluded that the variation could be explained by the methodology, that is, whether the study was in vitro or in vivo and the anatomical location of the skull examined.^9^ We have previously used data from this study^8^ to determine a cranial suture conductance of 0.32S/m using computational modeling.^10^

This present study aimed to demonstrate the importance of tailoring the modeled bone conductivity to better reflect the conductivity proximate to the electrode position and to investigate the effects of including the conductivity effects of cranial sutures in ROAST modeling of tES.

## METHODS

DICOM T1 MRI images and T2 MRI images were obtained from a 45-year-old male subject (AC) and were converted to NIFTI files. ROAST (version 3.0) was downloaded from https://github.com/andypotatohy/roast and files were integrated with MATLAB (version R2022a). tDCS simulation utilized the 10/05 electrode location system, which allowed flexibility to place the electrodes over the correct anatomical sites, including sutures and 1 mA of current applied between the anode and cathode. The 5-cm “disc” electrodes were applied to approximate the bitemporal (BT), bifrontal (BF), and right unilateral (RUL) montages used in ECT.

**FIGURE 1:**
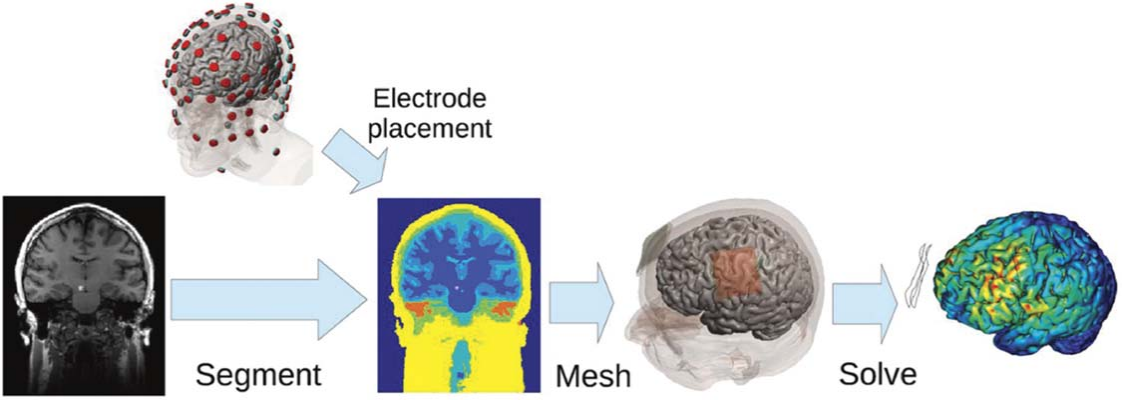
ROAST pipeline for building a current-flow model of the head. The input is the MRI of an individual and electrode placement and the output is the predicted electric field distribution. Each step: segmentation; meshing and solving are outlined in the methods section. This image is licensed under Creative Commons Attribution 3.0 license.

The ROAST pipeline is comprised of (see Fig. 1)^7^:

1. **Segment:** Segmentation is first done using the ‘Unified Segmentation’ algorithm implemented in Statical Parametric Mapping 12 (SPM12). It results in the following 6 tissue types: gray matter (GM), white matter (WM), cerebral spinal fluid (CSF), skull, scalp, and air cavities. The segmentation is further improved by Matlab script that smooths each of the tissues (using a Gaussian low-pass filter with *s mm* standard deviation), fills holes in CSF and removes disconnected voxels (using simple heuristics, for example, to fill gaps in CSF if any brain voxels touch the skull then these are convert to CSF). Of note, many of the small skull openings, including the cranial sutures are not preserved, only the optic canal and foramen magnum are preserved by making a special mask to mark these regions that were mapped onto the individual MRI space.
2. **Electrode placement:** using Matlab script and 10/05 electrode location system the shape of each electrode can be selected (disc, pad, or ring) and size and orientation adjusted.
3. **Mesh:** Finite element meshing is achieved using the Matlab toolbox, iso2mesh, with ‘cgalv2m’ function and a tetrahedral mesh is generated. The CGAL package generates a volumetric mesh from 3D multidomain images.
4. **Solve:** Finite element modeling (FEM) using the open-source getDP is used to solve the underlying Laplacian equation. Users can specify how much current is applied between the anode and cathode (Neumann boundary condition). The tissue conductivity can be customized with the default based on cited previous literature.

Computational modeling was first conducted with the default bone conductivity in ROAST (0.01S/m) then altered to account for electrode positioning on the temporal bone (0.0028S/m) or frontal bone (0.0126S/m).^8^ To simulate the impact of the sutures, pad electrodes, 0.1 mm high and 40 mm long, were applied to the scalp overlying the sutures and the bone conductivity was changed to 0.32S/m (our calculated suture conductivity^10^). The pads were correctly orientated and positioned so that they were directly over the squamous, coronal and sagittal sutures.

The electric field for our regions of interest was determined using a slice view in the model voxel space which allowed the determination of the electric field in V/m for a specific voxel with MNI coordinates demonstrated in a gray circle. The regions of interest selected were the subgenual anterior cingulate cortex (sgACC), given the antidepressant effects of ECT correlates with sgACC activity^11^ and the hippocampus (Hipp) given ECT induced changes have been correlated to cognitive side effects.^12^ The MNI coordinates used for the left and right “sgACC” was 154; 94; 163 and 154; 115; 163 respectively and for the left and right “Hip” was 124; 75; 133 and 124; 128; 133 respectively. Finally, high-resolution 3D rendered images were created for each tissue type.

T1 MRI DICOM images (HREC number HC190222 - Research MRI Scanning for protocol development) from the same subject were uploaded to “3D Slicer 5.0.2” and volume rendering was adjusted using the preset “CT-Fat,” and shift adjusted so the skull and sutures were visible to identify the squamous, coronal, and sagittal sutures (method previously described^10^).

## RESULTS

**FIGURE 2:**
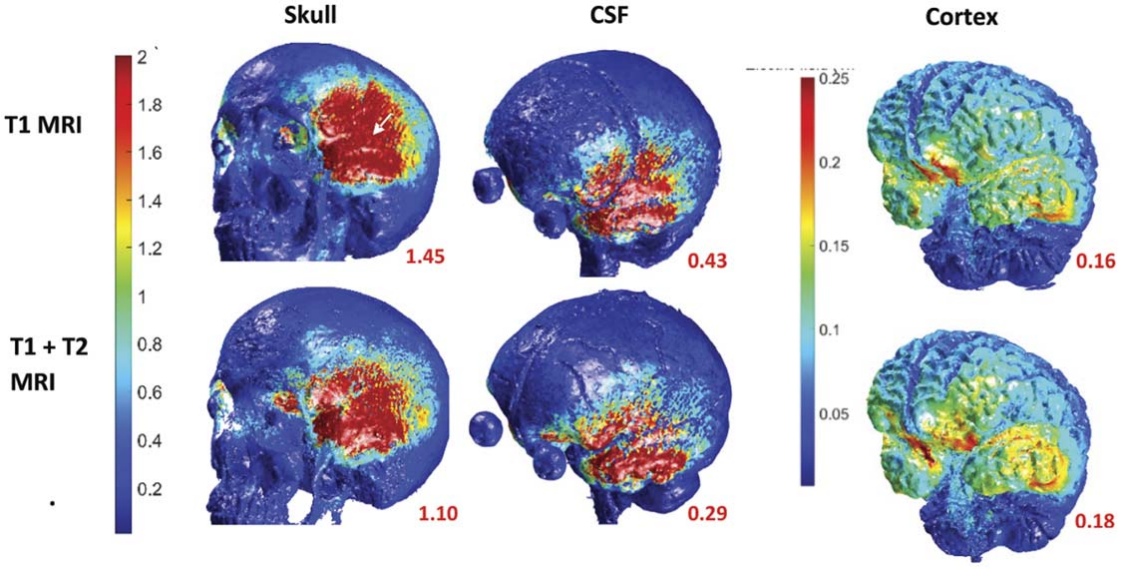
Comparing T1 MRI versus T1 and T2 MRI images to generate the head model in ROAST. A, Bitemporal tDCS montage (FFT9h left 5-cm disc anode and FFT10h right 5-cm disc cathode) with 1 mA and electric field (V/m) demonstrated on high resolution 3D renders of each tissue layer. The white arrow demonstrated the squamous suture. The red number next to each figure represents the maximum electric field (V/m) on the surface of each tissue layer.

**FIGURE 3:**
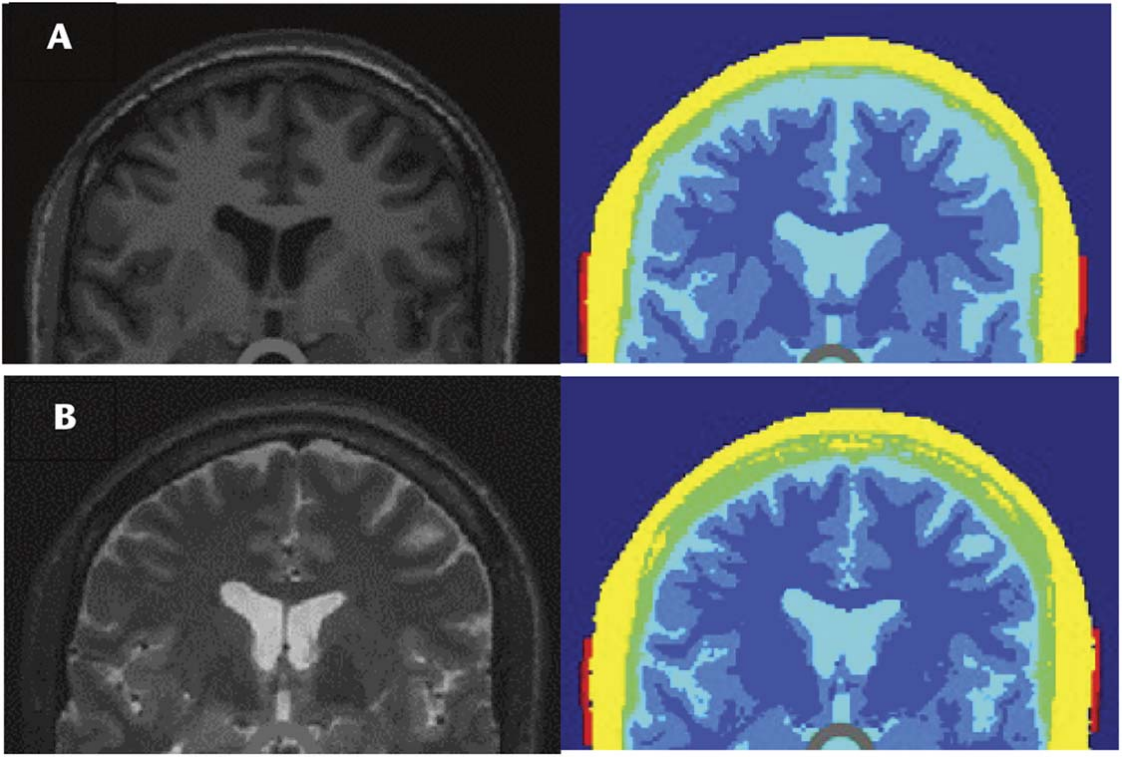
Coronal slice views of the (A) T1 MRI and (B) T1 and T2 MRI images and segmentation. The BT montage electrodes can be seen in red. The scalp (yellow), skull (green), CSF (cyan), gray matter (light blue), and white matter (dark blue) are demonstrated.

When using T1 MRI images for the ROAST modeling, T1 model, the squamous suture was clearly demonstrated in the external skull layer whereas in the T1 and T2 model the skull appeared rough over the frontal bone and the squamous suture was not visible (see Fig. 2). The scalp layer was also rough in the T1 and T2 model in contrast to the T1 model due to reduced contrast, between the scalp and air, in the raw MRI images, which resulted in less precise segmentation (see Fig. 3). Figure 3 demonstrates that the bitemporal electrodes in the T1 and T2 model do not make complete contact with the skin as compared with the T1 model. This resulted in a patchy electric field reaching the skull layer and a reduced intensity of the electric field (see Fig. 2). This reduced electric field continues to the CSF layer, which appears much smoother in the T1 and T2 model (see Fig. 2) and thinner (see Fig. 3) compared with the T1 model. Since less electric current is shunted away by the CSF, the electric field that reaches the cortex layer is higher. Given the T1 model was able to identify the squamous suture and the scalp layer was smoother, it permitted the use of very thin (0.1 mm) electrodes to simulate the cranial sutures, and thus the T1 model was selected for further simulations in this study.

**FIGURE 4:**
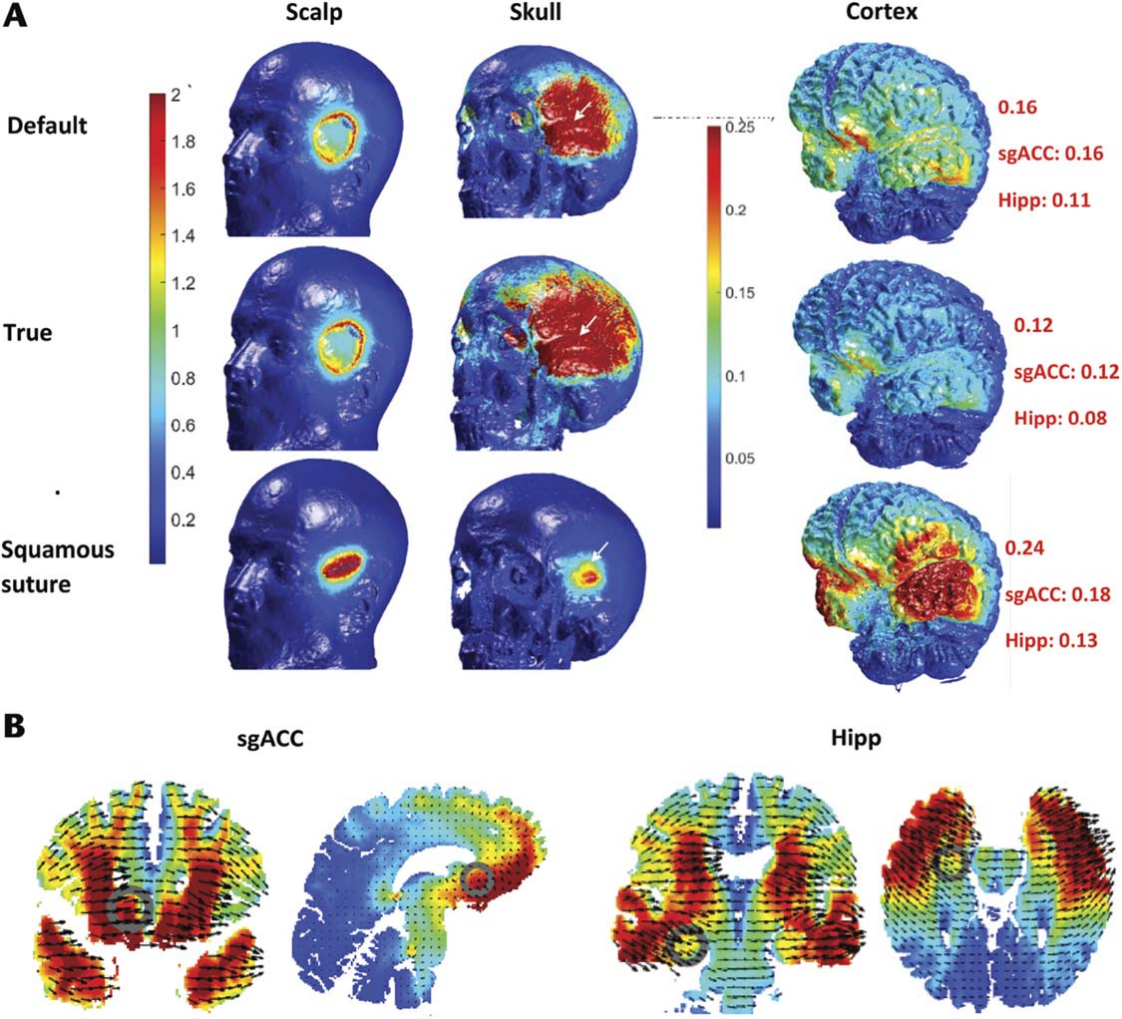
Bitemporal tDCS montage with skull conductivity set at default (0.01S/m) and true (0.0038S/m) and impact of squamous suture (SqS). A, BT montage (FFT9h left 5-cm disc anode and FFT10h right 5-cm disc cathode) with 1 mA and electric field demonstrated on high resolution 3D renders of each tissue layer. SqS was modeled using a pad electrode, 0.1 mm high and 40 mm long, orientated over the SqS (demonstrated by the white arrow) and the skull conductivity changed to conductivity of sutures, 0.32S/m. The red number represents the maximum electric field (V/m). B, Electric fields (in default model) over the subgenual anterior cingulate cortex (sgACC MNI: 154; 94; 163) and hippocampus (Hipp MNI 124; 75; 133) in coronal, sagittal, and axial slices.

**FIGURE 5:**
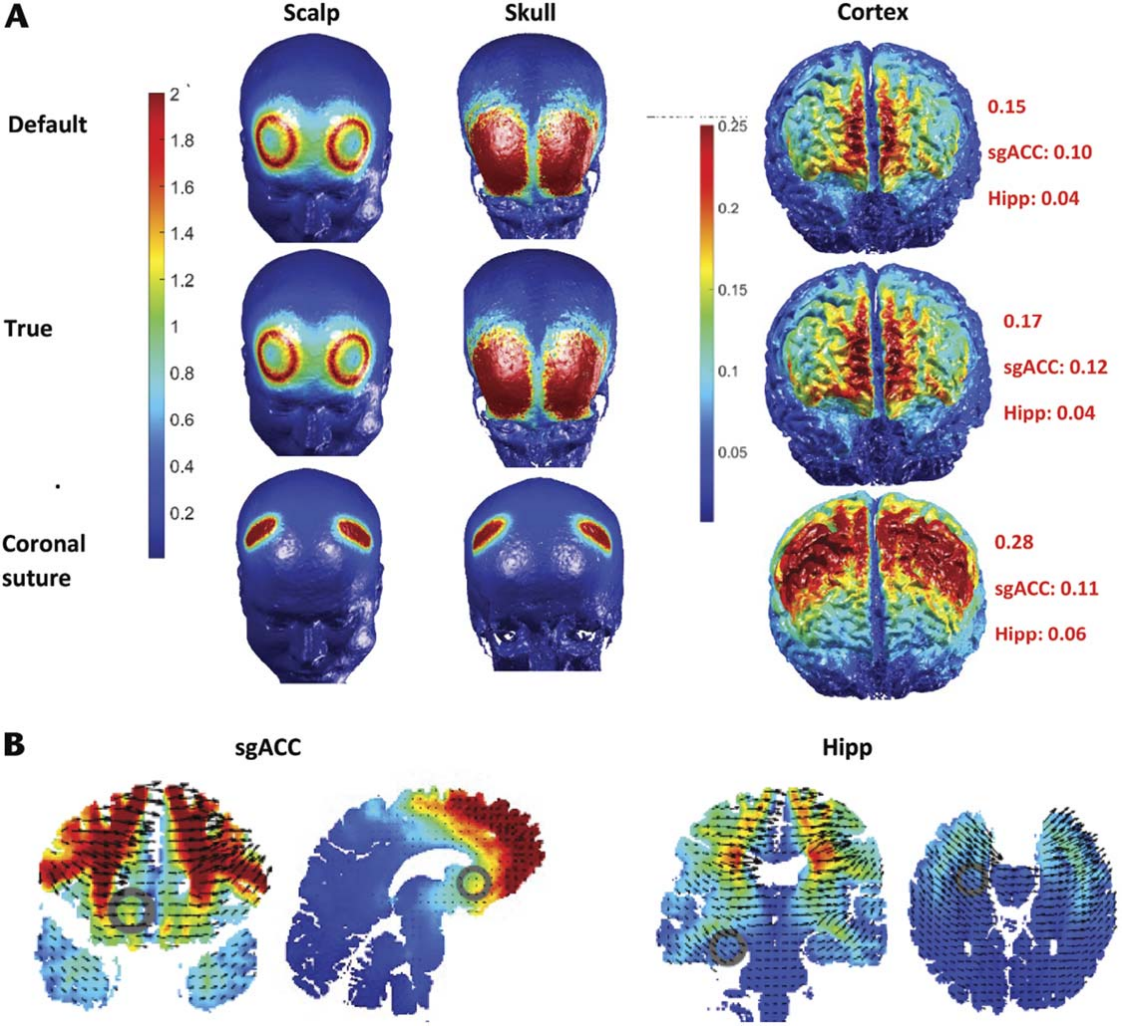
Bifrontal (BF) tDCS montage with skull conductivity set at default (0.1S/m) and true (0.0126S/m) and coronal suture (Cor) added (A) A BF montage (AF5h left 5-cm disc anode and AF6h right 5-cm disc cathode) with 1 mA and electric field demonstrated on high resolution 3D renders of each tissue layer. Cor was modeled using a pad electrode, 0.1 mm high and 40 mm long, orientated over the approximate position of the Cor and the skull conductivity changed to conductivity of sutures, 0.32S/m. B, Electric fields (in default model) over the subgenual anterior cingulate cortex (sgACC MNI: 154; 94; 163) and hippocampus (Hipp MNI 124; 75; 133) in coronal, sagittal, and axial slices.

Reducing the skull conductivity over the temporal bone from the default setting of 0.01S/m to 0.0038S/m,^8^ when simulating tDCS using a bitemporal montage, resulted in an increase in the electric field on the surface of the skull (area and maximum) and reduced the electric field on the cortex and deeper cortical structures (see Fig. 4). However, increasing the skull conductivity over the frontal bone from the default setting of 0.01S/m to the true conductivity of 0.0126S/m,^8^ when simulating tDCS using a bifrontal montage, had little impact (see Fig. 5).

**FIGURE 6:**
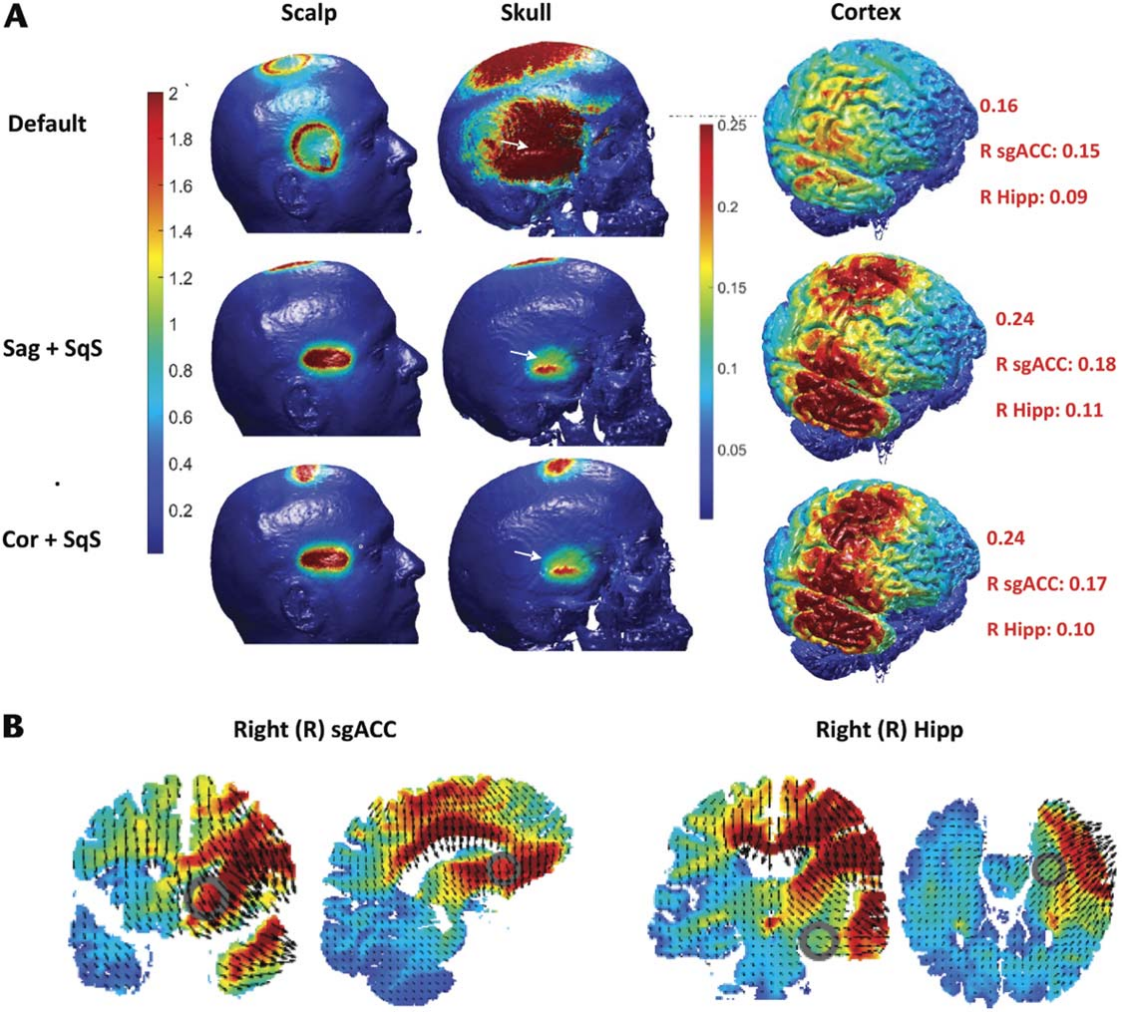
Right Unilateral (RUL) tDCS montage and impact of the squamous suture (SqS), sagittal suture (Sag) and coronal suture (Cor) (A) A RUL montage (FC2h right 5-cm disc anode and FFT10h right 5-cm disc cathode) with 1 mA and electric field demonstrated on high resolution 3D renders of each tissue layer. SqS (FFT10h), Sag (FCz), and Cor (FFC2h) were modeled using a pad electrode, 0.1 mm high and 40 mm long, orientated over the suture (SqS demonstrated by the white arrow) and the skull conductivity changed to conductivity of sutures, 0.32S/m. B, Electric fields (in default model) over the right subgenual anterior cingulate cortex (sgACC MNI: 154; 94; 163) and right hippocampus (Hipp MNI 124; 75; 133) in coronal, sagittal, and axial slices.

If cranial sutures had a conductivity of 0.32S/m^10^ the electric current would preferentially pass through these breaches in the highly resistant cortical bone of the skull. Simulating the sutures in the models resulted in a much higher electric field on the cortex and deeper cortical structures (see Figs. 4–6). The CSF layer has a higher conductivity and the current is shunted away from the target and this resulted in a greater area of electric field over the cortex.

**FIGURE 7:**
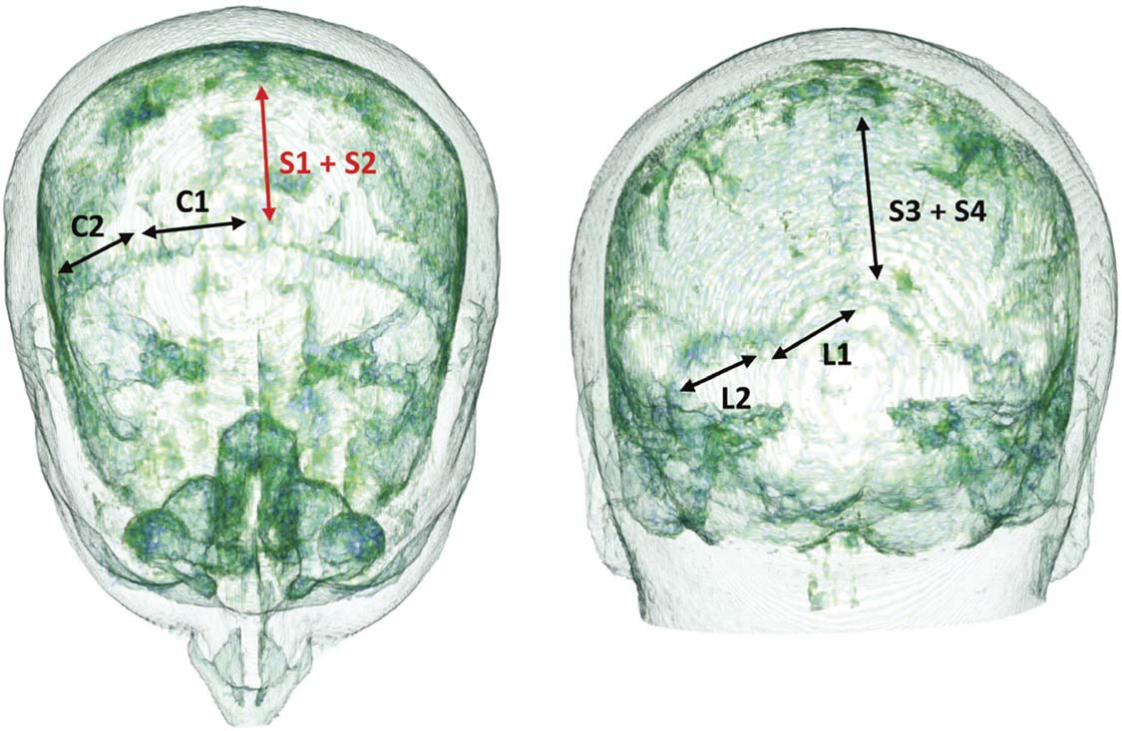
Volumetric rendering of T1 MRI images—DICOM images from same subject (AC) used in the ROAST models uploaded to “3D Slicer 5.0.2.” Volume rendering was shift adjusted so the skull and sutures were visible using the preset “CT-Fat.” C1 and C2 represent the coronal suture: first part and second part. S1, S2, S3, and S4 represent 4 parts of the sagittal suture and S1 and S2 have closed and not visible represented in red. L1 and L2 represent the lambdoid suture: first part and second part.

The squamous suture can easily be seen in the T1 Model (indicated by white arrows in Figs. 2, 4, and 6); however, the sagittal and coronal sutures are difficult to visualize. To verify the location of the sagittal and coronal sutures and to assess if they had closed, the same T1 MRI DICOM images were converted to a 3D image demonstrating the skull, sutures, and soft tissue through volumetric rendering using a method previously described.^10^ Figure 7 illustrates that the first and second parts of the coronal suture (C1 and C2) can be clearly seen (relevant to the modeling in Figs. 5, 6); however, the first 2 parts of the sagittal suture (S1 and S2) (relevant to the modeling in Fig. 6) cannot be seen and thus has likely closed whereas the third and fourth parts of the sagittal suture (S3 and S4) are still visible.

## DISCUSSION

This study aimed to demonstrate the importance of anatomically accurate head models in computational modeling of tES using ROAST. First, it showed that the T1 model, when compared to the T1 and T2 model, provided more precise scalp and skull tissue layers. In addition, the location of the squamous suture on the external skull was visible to allow for adjustment of the skull conductivity in this location and assess the impact. However, the CSF layer appeared much less smooth and was thicker in the T1 model, which would have resulted in a greater diffusion of electric field (given CSF is highly conductive) and reduced electric field maximum on the underlying brain cortex.

Varying the skull conductivity from the default setting in ROAST (0.01S/m) to conductivities based on conductivity in vivo^8^ had a large impact on the intracranial electric fields, when modeling the bitemporal montage but not in the case of the bifrontal montage. This is potentially due the bitemporal montage overlying the temporal bone, which lacks spongiform bone and leads to a much higher resistance to electrical current.

This study also demonstrated that the cranial sutures can be identified (squamous suture using the T1 model in ROAST and the sagittal and coronal sutures demonstrated in Fig. 7) and that including them in the ROAST T1 model increased both the intensity and the extent of the electrical field over the cortex and deeper cortical structures. This modeling has demonstrated that the cranial sutures act as foci of low resistance as the electric field from tES spreads across the highly resistant skull. Thus, prediction of electric flow should become more realistic if cranial sutures are incorporated.

Several limitations need to be acknowledged. Reliance on DICOM images from a single 45-year-old male subject may limit the generalizability of the findings given individual anatomical variations can significantly influence electrical field distributions. The ROAST pipeline did not allow the user to generate a mask for the cranial sutures; only the optic canal and foramen magnum are automatically included in the default setting. Other automated pipelines such as SimNIBS allow the user to add masks to the base model, which would result in more precise and reproducible modeling of tES. The study primarily focused on specific electrode montages: bitemporal, bifrontal and right unilateral, which are commonly used in ECT treatments; however, these do not encompass the full range of configurations used for tES in clinical practice and expanding the scope of montages used might provide a more comprehensive understanding of electric field dynamics. Finally, without direct in vivo validation of the simulation outcomes there is a risk of overfitting the customized conductivity values without considering variable conditions.

Future studies could utilize the SimNIBS pipeline, where a mask can be created representing all the sutures, which would allow many more montages that are clinically relevant to be investigated. For example, the posterior cerebellum has been targeted in tDCS studies to augment both motor and cognitive functioning.^13^ The lambdoid suture (see L1 and L2 in Fig. 7) is likely to influence the results of cerebellum stimulation given it could optimize the penetration of the weak tDCS current through the highly resistant skull. These computational models could be validated in vivo, using new MRI technology (MREPT) that acquires high resolution brain conductivity measurements^14^ while the tES is being conducted.

A study of 54 patients with late-life major depressive disorder undergoing ECT and longitudinal MRIs (both functional and structural) discovered decreased function in the superior orbitofrontal cortex led to both antidepressant response and cognitive impairment. Volume increases in the hippocampus were antidepressant-response specific and a decrease in amygdala and hippocampus volume was cognitive impairment specific. The group conducted computational modeling of the E-field and concluded that there was an optimal E-field required for neuroplasticity and clinical outcomes while not inducing cognitive dysfunction.^15^ However, the study did not consider the impact of cranial sutures on the E-field. Our group is currently conducting a blinded randomized study comparing different ECT montages, the RAFT ECT trial,^16^ where every patient has undergone a brain MRI and outcomes such as cognitive impairment could be assessed against individual variation in suture anatomy.

## References

[1] UK ECT Review Group . Efficacy and safety of electroconvulsive therapy in depressive disorders: a systematic review and meta-analysis. Lancet. 2003;361:799–808. 12642045 10.1016/S0140-6736(03)12705-5

[2] Sinclair DJM Zhao S Qi F . Electroconvulsive therapy for treatment-resistant schizophrenia. Schizophr Bull. 2019;3:730–732. 10.1093/schbul/sbz037 PMC6581135 31150556

[3] Lefaucheur JP Antal A Ayache SS . Evidence-based guidelines on the therapeutic use of transcranial direct current stimulation (tDCS). Clin Neurophysiol. 2017;128:56–92. 27866120 10.1016/j.clinph.2016.10.087

[4] Miranda PC Callejon-Leblic MA Salvador R . Realistic modeling of transcranial current stimulation: the electric field in the brain. Curr Opinion Biomed Eng. 2018;8:20–27.

[5] Bai S Gálvez V Dokos S . Computational models of bitemporal, bifrontal and right unilateral ECT predict differential stimulation of brain regions associated with efficacy and cognitive side effects. Eur Psychiatry. 2017;41:21–29. 28049077 10.1016/j.eurpsy.2016.09.005

[6] Nasimova M Huang Y . Applications of open-source software ROAST in clinical studies: a review. Brain Stimul. 2022;15:1002–1010. 35843597 10.1016/j.brs.2022.07.003 PMC9378654

[7] Huang Y Datta A Bikson M . Realistic volumetric-approach to simulate transcranial electric stimulation-ROAST-a fully automated open-source pipeline. J Neural Eng. 2019;16:056006. 31071686 10.1088/1741-2552/ab208d PMC7328433

[8] Tang C You F Cheng G . Correlation between structure and resistivity variations of the live human skull. IEEE Trans Biomed Eng. 2008;55:2286–2292. 18713698 10.1109/TBME.2008.923919

[9] McCann H Pisano G Beltrachini L . Variation in reported human head tissue electrical conductivity values. Brain Topogr. 2019;32:825–858 Epub 2019 may 3. Erratum in: Brain Topogr 2021 Jan;34(1):110–115. PMID: 31054104. 31054104 10.1007/s10548-019-00710-2 PMC6708046

[10] Carroll A Rae CD Martin D . The effect of cranial sutures should be considered in transcranial electrical stimulation. J ECT. 2024. 10.1097/YCT.0000000000001079 PMC12105966 39652012

[11] Liu Y Du L Li Y . Antidepressant effects of electroconvulsive therapy correlate with subgenual anterior cingulate activity and connectivity in depression. Medicine (Baltimore). 2015;94:e2033. 26559309 10.1097/MD.0000000000002033 PMC4912303

[12] Argyelan M Lencz T Kang S . ECT-induced cognitive side effects are associated with hippocampal enlargement. Transl Psychiatry. 2021;11:516. 34625534 10.1038/s41398-021-01641-y PMC8501017

[13] Oldrati V Schutter DJLG . Targeting the human cerebellum with transcranial direct current stimulation to modulate behavior: a meta-analysis. Cerebellum. 2018;17:228–236. 28786014 10.1007/s12311-017-0877-2 PMC5849643

[14] Cao J Ball I Humburg P . Repeatability of brain phase-based magnetic resonance electric properties tomography methods and effect of compressed SENSE and RF shimming. Phys Eng Sci Med. 2023;46:753–766. 36995580 10.1007/s13246-023-01248-1 PMC10209292

[15] Qi S Calhoun VD Zhang D . Links between electroconvulsive therapy responsive and cognitive impairment multimodal brain networks in late-life major depressive disorder. BMC Med. 2022;20:477. 36482369 10.1186/s12916-022-02678-6 PMC9733153

[16] Loo C Barreiros AR Martin D . A randomized controlled trial of ultrabrief right unilateral ECT with frontoparietal versus temporoparietal electrode placement for severe depression-the RAFT ECT trial. J ECT. 2024;40:229–231. 38968425 10.1097/YCT.0000000000001018

